# Mitral prosthetic size predictor in minimally invasive mitral valve replacement

**DOI:** 10.1186/s13019-020-01197-w

**Published:** 2020-06-18

**Authors:** Anh T. Vo, Nguyen T. H. Nguyen, Khoi M. Le, Nguyen L. Vuong, Trang T. T. Nguyen, Thanh T. Vu, Sy V. Hoang, Dinh H. Nguyen

**Affiliations:** 1 Cardiovascular surgery department, University Medical Center, University of Medicine and Pharmacy at Ho Chi Minh city, 215 Hong Bang St – District 05, Ho Chi Minh City, Vietnam; 2 Department of thoracic and Cardiovascular Surgery, Faculty of Medicine, University of Medicine and Pharmacy at Ho Chi Minh city, Ho Chi Minh City, Vietnam; 3 Department of Medical Statistics and Informatics, Faculty of Public Health, University of Medicine and Pharmacy at Ho Chi Minh city, Ho Chi Minh City, Vietnam; 4 Department of Internal Medicine, Faculty of Medicine, University of Medicine and Pharmacy at Ho Chi Minh city, Ho Chi Minh City, Vietnam

**Keywords:** Prosthetic size predictor, Annular rupture, Minimally invasive approach, Cardiac computed tomography, Transthoracic echocardiography

## Abstract

**Background:**

Minimally invasive mitral valve replacement has become popular across the world. However, annular rupture and patient – prosthetic mismatch (PPM) are still problematic, particularly in the Asian population. To avoid this, a predictor model could be beneficial. Our study aimed to assess the value of mitral valve diameters measured on TTE and CT scan on predicting the actual mitral prostheses.

**Methods:**

From January 2018 to December 2019, a total number of 96 patients underwent minimally invasive mitral valve replacement. The association between imaging measurements and the outcome was checked by scatter plot and Pearson’s correlation coefficient. Univariable linear regression was used to build the prediction model.

**Results:**

The three strongest correlations for the whole population are the following features: Mean TTE diameter (0.702), mean diameter on CT lowest plane through the mitral annulus (0.679), and area-derived diameter on CT highest plane through the mitral annulus (0.665). The prosthetic size of the tissue valve group was more correlated to the calculated annulus diameters than that of the mechanical valve group. Tissue valve size predictor models based on these calculated diameters were 16.19 + 0.27 × d (r = 0.744), 12.74 + 0.44 × d (r = 0.756) and 12.79 + 0.38 × d (r = 0.730), respectively.

**Conclusion:**

Mitral prosthetic size could be predicted based on the mitral diameters measured on TTE and CT scan. The overall correlation coefficient varied from 0.665 (CT Scan) to 0.702 (TTE). These models performed better when applied to bioprosthesis.

## Introduction

Mitral valve disease is a common valvular heart disease, which includes mitral stenosis, mitral regurgitation and the combination of both conditions. In addition to valve repair, mitral replacement is an important alternative treatment. Moreover, the minimally invasive approach via right minithoracotomy has become popular across the world, owing to many advantages for patients such as less pain and bleeding, shorter intensive care unit (ICU) time and more satisfying cosmetic results [1]. However, annular rupture and patient – prosthetic mismatch (PPM) are still problematic and may affect short-term as well as long-term outcome after mitral valve replacement, particularly in the Asian population [2]. A good predictor of prosthetic size before operation should be performed to minimize the problem. The prediction could be done based on the preoperative imaging modalities including transthoracic echocardiography (TTE) and cardiac computed tomography scan (CT Scan). Some authors have addressed this issue before [3, 4]. However, these articles were conducted for more than a decade and there is still a lack of consistent models. Therefore, the purpose of this study is to assess the value of mitral valve diameters measured on TTE and CT scan on predicting the actual mitral prostheses.

## Material and methods

From January 2018 to December 2019, we investigated all patients undergoing minimally invasive mitral valve replacement via a right minithoracotomy at the University Medical Center, University of Medicine and Pharmacy at Ho Chi Minh City, Viet Nam. Patients with more than moderate aortic regurgitation, a history of right chest surgery or chest irradiation, severe aortoiliac stenotic diseases and prior cardiac surgery were excluded. Perioperative data was collected prospectively and analysed. A preoperative cardiac 128 – slice CT scan and TTE were performed in all patients. The mitral annulus parameters from these two imaging modalities were measured.

The mitral annulus does not have a planar configuration. It has a well-known saddle shape with the lowest plane consisting of points locating at the fibrous trigone, and the highest points locating at the midpoints of the anterior and posterior annuli [5]. Based on this fact, we assumed that the mitral annulus could not be accurately measured by a unique imaging method and so it has to be a combination of multiplanar measurements.

**Fig. 1:**
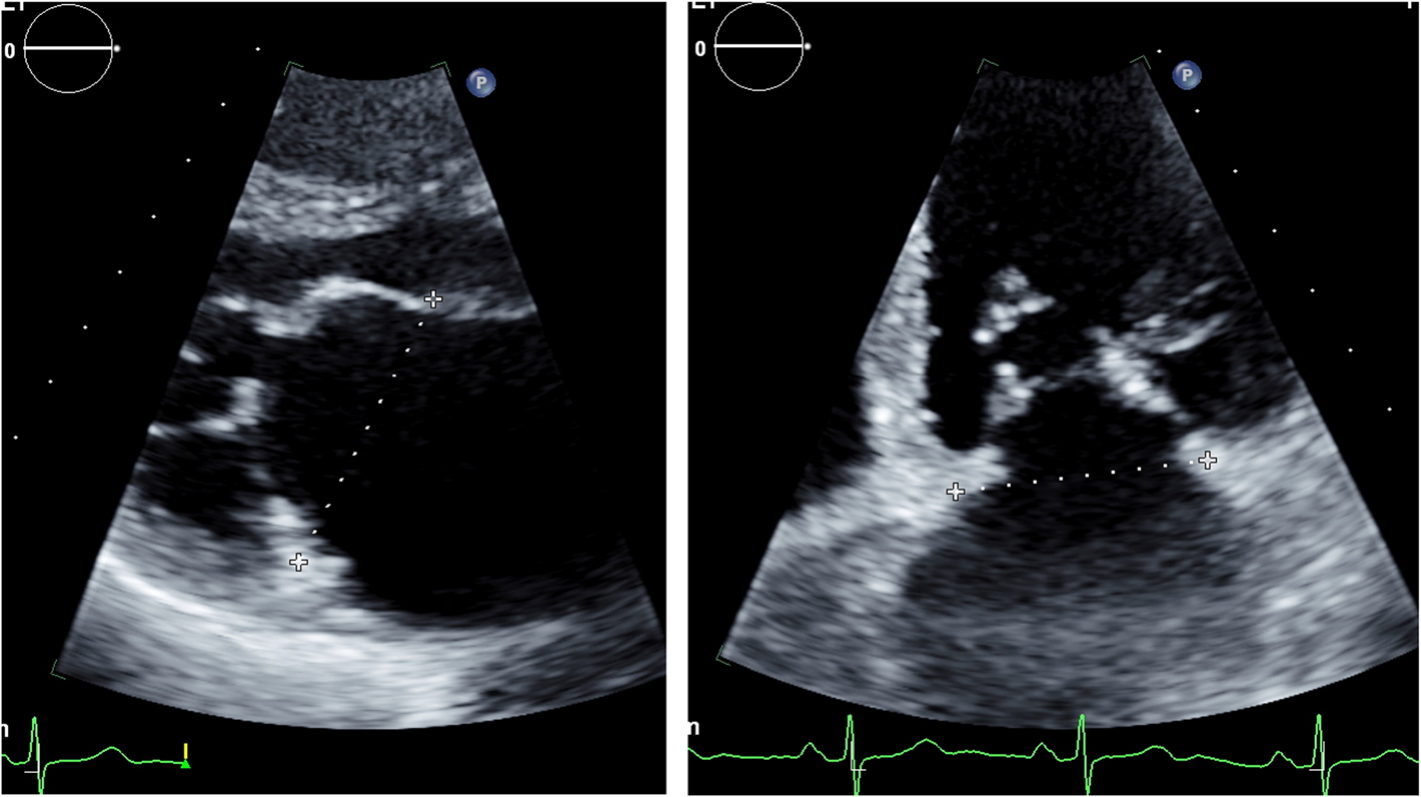
Mitral valve annulus measurements on TTE: PLAX (left) and A4C (right)

On TTE, we chose the two popular views to measure the mitral annular diameter: The apical 4 chamber (A4C) view and the parasternal long axis (PLAX) view. The average value of these two diameters constitutes the estimated diameter of the mitral valve annulus on TTE (Fig. 1).

**Fig. 2:**
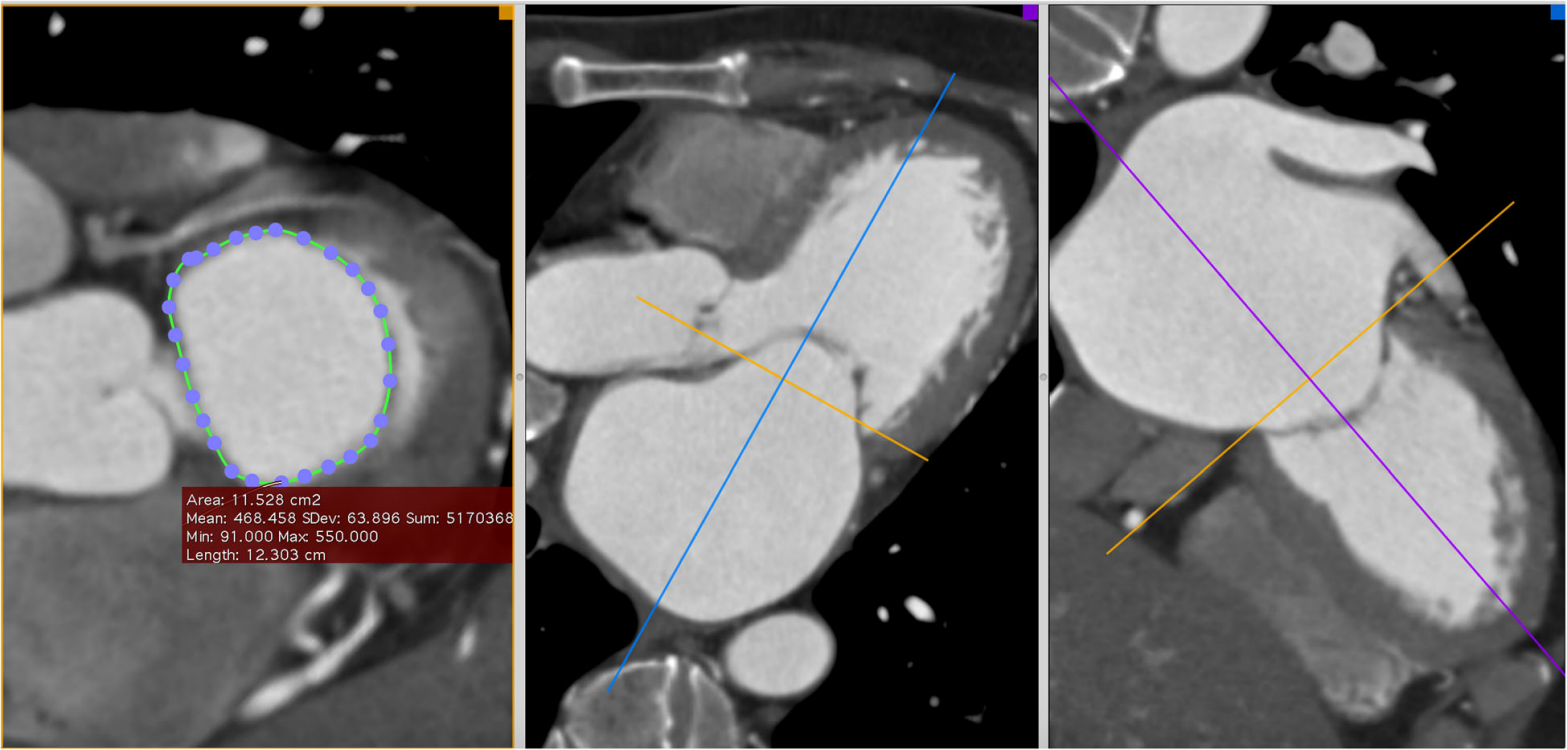
Mitral annulus area and perimeter measured at the lowest plane (passes through two trigones)

On CT Scan, by using multiplanar reconstruction via the OsiriX™ software (Bernex, Switzerland), we recreated the highest and the lowest planes of the mitral annulus. From each plane, we measured the smallest and the largest diameter and calculated the average diameter. The perimeter-derived diameter and the area-derived diameter were also determined. As a result, three different diameters for each plane were calculated (Fig. 2).

All diameters were measured at the end of the diastolic phase.

### Surgical technique

The patient was placed on the supine position with a cushion under the right shoulder. Cardiopulmonary bypass (CPB) was set up with cannulation of the right femoral vessels. A 5 cm skin incision was made parallel to the anterior axillary line and a video camera was inserted through a 5 mm port in the third right intercostal space. The Chitwood aortic cross-clamp was inserted and aortic clamping was performed. Custodiol HTK solution was delivered antegradely into the aortic root and was repeated every 120 min if necessary. A left atriotomy is performed and a left atrial retractor was used to expose the mitral valve. We then assessed the mitral valve for the feasibility of repairing. In this series, hence, mitral valve replacement was performed with a mechanical valve or a tisse valve. We preserved the posterior leaflet whenever possible, even in rheumatic patients. Pledgeted sutures were placed on the atrial side of the annulus and the valve was replaced in usual manners. Transesophageal Echocardiography (TEE) was used to control the result of the operation.

We used SJM™ Masters Series for mechanical valve (Abbott Laboratories, Chicago, Illinois, USA) and Carpentier-Edwards Perimount Plus and Perimount Magna Ease (Edwards Lifesciences, Irvine, California, USA) for tissue valve.

### Data analysis

Statistical analysis was performed using statistical software R version 3.6.1. Firstly, we checked the association between each imaging measurement with each other and with the outcome (valve size) by scatter plot and Pearson’s correlation coefficient. Linear regression model was used to predict the outcome from the measurements. Because of the strong correlation between parameters, we performed univariable linear regression to build the prediction model (outcome = a + b x measurement; a: intercept; b: slope). We chose three measurements which had the strongest correlation with the outcome (i.e., the highest correlation coefficient) to build prediction model. To predict the valve size preoperatively, 95% confidence interval (CI) of the slope and the intercept from the models were taken into account. The whole population was analysed first, then the tissue valve group and the mechanical valve group separatedly.

Our data was part of a research approved by the ethical board of the University of Medicine and Pharmacy at Ho Chi Minh City, number 141/DHYD-HDDD, on April 112,018.

## Results

From January 2018 to December 2019, a total of 96 patients underwent minimally invasive mitral valve replacement via a right minithoracotomy. Baseline patients’ characteristics are shown in Table 1. The mean age was 54.2 and males were slightly more numerous than females (51 vs. 45 cases). There were 39 patients (40.6%) with post rheumatic disease and 57 (59.4%) with degenerative disease. The number of cases replaced with mechanical and tissue valves were 46 (47.9%) and 50 (52.1%), respectively. The mean prosthetic size was 26.8 mm. Most of the posterior leaflets (94/96 cases, 97.9%) were preserved. One patient died in the ICU due to delayed annular rupture. The surgical characteristics and early postoperative complications are included in Table 2. At 6- month follow-up, no mortality and reoperations were recorded.

**Table 1:** Baseline patients’ characteristics

Variables	Number (***n*** = 96)
Age (years), mean ± SD	54.2 ± 9.5
Male, n (%)	51 (53.1)
BMI	21.9 ± 6.8
Comorbidities, n (%)	
+ Hypertension, n (%)	42 (43.8)
+ Type II diabetes mellitus, n (%)	27 (28.1)
+ Preoperative atrial fibrillation, n (%)	31 (32.3)
Etiology	
+ Post rheumatic, n (%)	39 (40.6)
+ Degenerative, n (%)	57 (59.4)
TTE characteristics	
Mean ejection fraction (EF, %)	58.4 ± 11.2
Mitral regurigation	57
Mitral stenosis	23
Mitral stenosis and regurgitation	16
NYHA classifications	
+ NYHA I, *n* (%)	7 (7.3)
+ NYHA II, *n* (%)	79 (82.3)
+ NYHA III, *n* (%)	9 (9.4)
+ NYHA IV, *n* (%)	1 (1)
Types of prosthesis	
+ Mechanical valve, *n* (%)	46 (47.9)
+ Tissue valve n (%)	50 (52.1)
Valve size (mm), mean ± SD	26.78 ± 3.36
+ Mechanical valve	26.91 ± 3.4
+ Tissue valve	26.67 ± 3.26
Posterior leaflet preservation, n (%)	94 (97.9%)

**Table 2:** Surgical characteristics and early postoperative complications

Surgical characteristics	
Cross-clamped time (minutes)	68.2 ± 11.7
Cardiopulmonary bypass time (min)	97.7 ± 19.6
ICU time (hours)	18.6 ± 10.3
Mean early postoperative transmitral gradient (mmHg)	3.4 ± 1.7
Early complications	
+ 30-day mortality, n (%)	1 (1.04)
+ Heart failure required ECMO, n (%)	0 (0)
+ Renal failure required dialysis, n (%)	0 (0)
+ Pneumonia, n (%)	5 (5.2)
+ Reoperation due to bleeding, n (%)	3 (3.1)
+ Annular rupture, *n* (%)	1 (1.04)
+ Myocardial infarction, *n* (%)	0 (0)
+ Femoral artery stenosis, *n* (%)	1 (1.04)
+ Conversion to sternotomy, *n* (%)	2 (2.1)

**Fig. 3:**
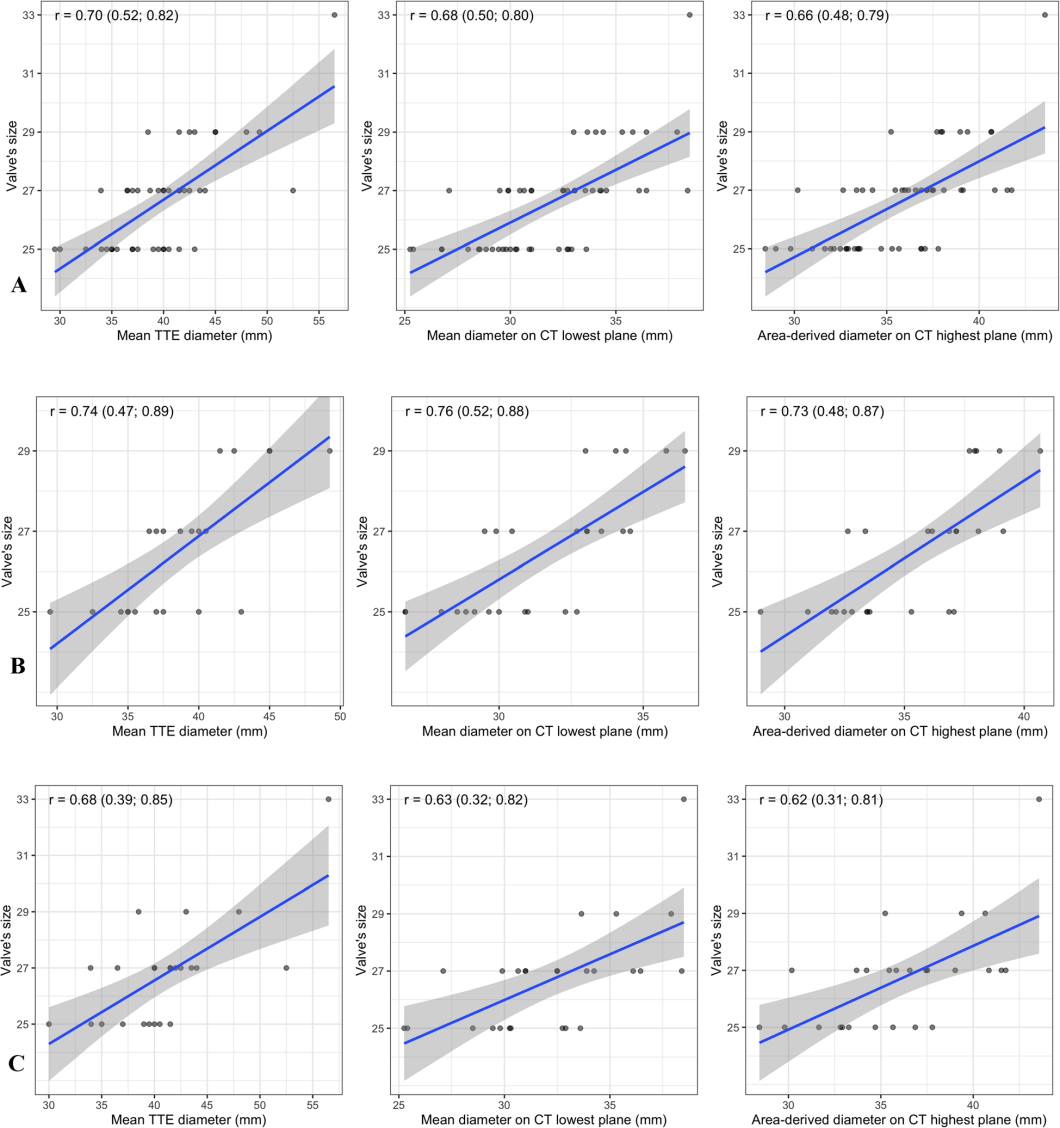
Scatterplots for the correlation of each calculated diameters with prosthetic size: **a** Whole population, **b** tissue valve group, **c** mechanical valve group

According to the analysis, the three strongest correlation for the whole population are:

- Mean TTE diameter (0.702)
- Mean diameter on CT lowest plane (0.679)
- Area-derived diameter on CT highest plane (0.665) (Fig. 3).

The prosthetic size of the tissue valve group seemed to be more correlated to the calculated annulus diameters than that of the mechanical valve group (Table 3).

**Table 3:** Correlation coefficient between calculated diameter and the prosthetic siz**e**

Calculated mitral annulus diameters	All patients	Mechanical valve	Tissue valve
Mean TTE diameter (PLAX diameter and A4C diameter)	0.702	0.683	0.744
Lowest plane on CT scan (passed through the trigones)			
Mean diameter	0.679	0.629	0.756
Perimeter-derived diameter	0.650	0.628	0.683
Area-derived diameter	0.663	0.639	0.695
Highest plane on CT Scan (passed through midpoints of the anterior and posterior annuli)			
Mean diameter	0.587	0.522	0.682
Perimeter-derived diameter	0.622	0.575	0.704
Area-derived diameter	0.665	0.622	0.730

### Univariable linear regression prediction models for mitral prosthesis size

We used the three highest correlated calculated diameters to build prediction model for the mitral prosthesis size. Table 4 showed the formulae for the whole population, the tissue valve group and the mechanical valve group.

**Table 4:** Prediction model for mitral prosthesis size

Calculated diameter	Whole population	Mechanical valve group	Tissue valve group
Mean TTE diameter	17.28 + 0.23 × d (*R*^2^ = 0.493)	17.51 + 0.23 × d (*R*^2^ = 0.464)	16.19 + 0.27 × d (*R*^2^ = 0.554)
Mean diameter on CT lowest plane	15.09 + 0.36 × d (*R*^2^ = 0.461)	16.39 + 0.32 × d (*R*^2^ = 0.396)	12.74 + 0.44 × d (*R*^2^ = 0.572)
Area-derived diameter on CT highest plane	14.88 + 0.33 × d (*R*^2^ = 0.442)	16.10 + 0.29 × d (*R*^2^ = 0.386)	12.79 + 0.38 × d (*R*^2^ = 0.533)

*d* corresponding calculated diameter; *R*^2^ R-squared of the model

## Discussion

Mitral valve disease comprises of mitral regurgitation, mitral stenosis and the combination of these two entities. Surgery remains a cornerstone in the treatment of the disease. For the last 2 decades, minimally invasive approach via a right minithoracotomy has become a new trend for mitral valve surgery, with many advantages, namely less pain and blood loss, shorter intensive care unit (ICU) time and more satisfying cosmetic outcomes [1]. Mitral valve repair has been proven to have a good short-term and long-term results in comparison with mitral replacement, particularly in degenerative etiology [6]. However, rheumatic mitral valve remains a problem in developing countries with a prevalence approximating 5.5–5.7/1000 [7], as more than 40% of our patients are post rheumatic. This condition, along with complex degenerative mitral lesion, is making mitral valve replacement irreplaceable.

The rheumatic lesion in combination with the low body mass index (BMI) in Asian people might lead to a small mitral annulus, thus creating a technical risk of annular rupture in mitral valve replacement, which is a fatal complication. The incidence of left ventricular rupture after mitral valve replacement is roughly 1.2% and the mortality could reach up to 70% [8]. It requires sternotomy conversion and results in high mortality, particularly in minimally invasive mitral surgery. We experienced one case of mitral annulus rupture while performing valve replacement via the minimally invasive approach, this has been reported elsewhere [9]. Zhai et al. also reported two cases of cardiac rupture in a population undergoing minimally invasive mitral surgery, both patients died [10]. On the other hand, PPM was also reported in patients with mitral valve replacement. In the mitral valve position, PPM is diagnosed when the effective orifice area index (EOAI) is ≤1.2 to 1.25 cm^2^/m^2^. This condition could have the same pathophysiology with mitral stenosis, resulting in dilation of the left atrium, pulmonary hypertension and right heart failure [11]. Furthermore, PPM can significantly affect long-term outcomes of mitral valve replacement [2].

These morbidities hence demand a preoperative prediction of the prosthetic size to anticipate the intraoperative risks. Decades ago, prediction models based on body surface area (BSA) have been established. However, these models were proven to be inaccurate and were not helpful for surgeons in the predetermination of the size of the mitral prostheses [3]. Caldwell et al. reported the role of 2D TTE in predicting the size of aortic and mitral protheses in children and concluded that this method was useful in primary valve replacement [4]. Nevertheless, the method has become obsolete for years, being mentioned by very few authors. Nowadays, with the support of advanced cardiac imaging modalities, including high resolution TTE and ECG-gated CT Scan, the need of an effective predictor model has returned.

Our results showed a fairly good correlation between the diastolic diameters of the mitral annulus, measured by TTE and CT Scan and the prosthetic size, with the best coefficient value being 0.702. This indicates a strong positive linear relationship between the predict diameters and the valve size. Unlike our anticipation, echocardiography performs surprisingly better than CT Scan in predicting the mitral prosthetic size with the correlation coefficient of 0.702. The coefficient value of multiple diameters on CT Scan measured on the two planes did not exceed 0.70. The best correlated values of CT Scan measurements are the mean diameter on CT scan lowest plane through the annulus and the area-derived on CT scan highest plane through the annulus, with the values of 0.679 and 0.665, respectively.

Before the analysis, we expected that CT scan would become a good modality for predicting the prosthetic size, thanks to the flexibility of 3D reconstruction of the mitral planes. Nevertheless, the simple but effective TTE seemed to be better than multiplanar reconstruction CT Scan in this field. To explain this, we assumed that the limitation of CT Scan was the incapability of capturing the heart throughout an entire cardiac cycle. It produced a static image of the heart and did not grant a choice for us to pick up another option if the illustration was not optimal. While CT scan is very dedicated in detecting small structural abnormalities, there are still notable limitations. It only provides good quality images of static structures. The image can easily be blurry if the studied organ moves irregularly, as seen in atrial fibrillation. On the other hand, high resolution TTE provided a full animation of the cardiac cycle, and the physicians had the ability to choose the ideal images of the mitral annulus in PLAX and A4C views.

When analysed separately, our predictor models performed better for tissue valves than for mechanical valves. With bioprosthesis, the three models showed a strong relationship between the calculated diameters and the prosthetic size. In contrast, the coefficient of these models decreased to less than 6.5 when applied to mechanical valve. The reason for this difference could be explained by the etiology of mitral valve disease. Mechanical valves were mostly used in young patients, who usually suffered from rheumatic heart disease. The deformity of the rheumatic mitral annulus is complicated, it tends to be smaller, round, calcified and stiffer, along with multiplanar asymmetrical dilatation [5]. These changes made the measurements less correlative with the prosthetic size. In contrast, tissue valves were frequently replaced in older population, in whom degenerative disease is dominant. The degenerative mitral annulus is usually dilated and flattened [12, 13], making it more similar to the prothesis’ single planar configuration. As a result, the measurements on CT scan and TTE become closer to the true value. This would subsequently increases the accuracy of the predicting models. For these reasons, we recommend using these models on degenerative mitral valve disease, particularly when a tissue valve is needed.

### Limitations

The number of patients of this series is limited, making the conclusion less significant. Errors in measuring was reduced with only one physician doing the measurements, however it could not be excluded, and its impact could not be measured exactly due to small sample size.

## Conclusion

Mitral prosthetic size could be predicted based on the measured mitral diameters on TTE and CT scan. The overall correlation coefficient varied from 0.665 (CT Scan) to 0.702 (TTE). These models performed better when applied to bioprosthesis. The three best diameters for the predictions are mean diameter on TTE, mean diameter on CT lowest plane of the mitral annulus and area-derived diameter on CT highest plane of the mitral annulus.

## Data Availability

The datasets used and/or analysed during the current study are available from the corresponding author on reasonable request.
